# Pairing taguchi based design of experiment with response surface methodology for diesel engine performance optimization using biodiesel-magnesium oxide nanoparticles blends

**DOI:** 10.1371/journal.pone.0341542

**Published:** 2026-03-04

**Authors:** Muhammad Usman Zafar, Heba G. Mohamed, Khaled Alnamasi, Abdullah M.A. Alsharif, Muhammad Nasir Bashir

**Affiliations:** 1 Department of Mechanical Engineering, University of Engineering and Technology Lahore, Pakistan; 2 Department of Electrical Engineering, College of Engineering, Princess Nourah bint Abdulrahman University, Riyadh, Saudi Arabia; 3 Department of Mechanical Engineering, College of Engineering, King Faisal University, Al-Ahsa, Saudi Arabia; 4 Department of Mechanical Engineering, College of Engineering at Yanbu, Taibah University, Yanbu Al-Bahr, Saudi Arabia; 5 Multi-Scale Fluid Dynamics Lab, Department of Mechanical Engineering, Yonsei University, Seoul, Republic of Korean; Vietnam Maritime University, VIET NAM

## Abstract

The increasing demand for diesel engines in agriculture, transportation and power generation has led to the overconsumption of fossil fuels, demanding the search for sustainable alternatives. Biodiesel has emerged as the promised alternative as it offers environmental and economic benefits. This study explores the impact of magnesium oxide (MgO) nanoparticles as an additive to the biodiesel-diesel blends on diesel engine performance. Experimental investigations were conducted on four-cylinder diesel engines under varying engine speeds, load conditions, biodiesel blends and magnesium oxide concentrations. A Taguchi L_18_ orthogonal array and response surface methodology (RSM) was employed for optimal brake-specific fuel consumption (BSFC) and brake thermal efficiency (BTE). Using Taguchi paired RSM method, three input factors – Biodiesel percentage in diesel (0%, 10%, 20% v/v), engine load (25%, 50%, 75%) and MgO nanoparticles dosage of (0 g, 0.02 g, 0.04g) – were varied to assess the influence on the Brake Specific fuel consumption and Brake Thermal Efficiency. Results indicated that the addition of the MgO nanoparticles enhances combustion efficiency resulting in increase in BTE while decrease in BSFC. The maximum BTE of 23.54% was obtained at the biodiesel percentage of 12%, speed of 1200 RPM, 75% load, and 0.04g MgO. The minimum BSFC was 306.983 g/kWh was obtained at 8% biodiesel and 0.04g MgO, operating at the speed of 1200 RPM and 25% load on engine. The theoretical error in maximum BTE and minimum BSFC as compared to experimental was 1.12% and 4.6% respectively. Optimal engine performance was observed at the moderate biodiesel blends ranging between 12–16 percent with MgO at 0.04g and running between the speeds of 800–1600 RPMs. Using RSM, 7% of cost savings were obtained for the optimal cases as compared to adverse conditions in Automotive industry and 5% savings was obtained for the heavy-duty industry. These findings conclude that biodiesel blends can improve the thermal efficiency and fuel economy. The use of Taguchi L18 provides robust and low-cost procedure for the performance analysis of the diesel engine.

## 1. Introduction

The usage of petroleum derivatives has grown steadily due to increase in population and industrialization. Diesel engines are popular for their high conversion efficiency and reliability [1]. Due to these characteristics, diesel engines are used in automotive, power generation, agriculture and shipping sectors. The extensive usage of the diesel engine has caused overconsumption of the fossil fuels. There is a looming shortfall of fossil fuels forecasted by mid- 21^st^ century [2,3]. In response to these challenges, clean and cost-effective alternatives to diesel take a paramount importance in addressing fuel crisis. The rapid depletion of the crude oil supply has made it important to adapt the usage of biodiesel, a financially stable and promising alternative to the diesel. Many studies have confirmed that biodiesel is an inexpensive, environmentally friendly and easily accessible source. Several researches have been done over the effects of different raw materials for bio diesel engine [4].

BTE expresses the fuel-to-air ratio ability to produce work in an engine by showing how efficiently the energy in fuel is transformed into useful work [5]. The use of such fuels is not without drawbacks. It must be noted that production and distribution infrastructure that corresponds to the widespread use of these fuels must be created and advanced [6]. Besides, the cost of other energy sources is expensive compared to conventional hydrocarbon energy sources. But ongoing studies have shown that with increased effort in research and development, the costs are bound to come down and making the use of different forms of fuels commercially viable [7].

Biodiesel can be used in the diesel engines without any engine alterations. Biodiesel has high flash point, high oxygen content and better combustibility than conventional diesel fuel [8]. Ellappan et al. [9] showed that biodiesel blends have high values of BTE than that of conventional diesel. The blends had better combustibility owing to the presence of a higher percentage of oxygen in biodiesel. Despite these advantages, the adoption of biodiesel has faced several challenges with engine performance. A challenge in adoption of biodiesel is the incomplete combustion of the fuel in the engine [10]. This has led the researchers to explore various strategies regarding manipulation of fuel properties to suit diesel engine performance [11]. Various fuel additives and enhancements, including nanoparticles (NPs), alcohols and aromatic compounds have been explored. In this matrix of additives, the NPs have got considerable attention due to their catalytic characteristics and heat transfer capabilities. Among different kinds of the NPs, metal and metal oxide particles have gained popularity due to their high heat transfer and excellent catalytic properties. Sharifianjazi et al. [5] showed that biodiesel blends with ${Al}_{\text{2}}O_{\text{3}}$ nanoparticles led to enhanced BTE. The study revealed that the catalytic properties of ${Al}_{\text{2}}O_{\text{3}}$ caused much better combustion. Leo et al. [12] observed that the utilization of metal oxide nanoparticles in biodiesel blends enhanced the BTE that described the catalytic effectiveness of metal oxide nanoparticles.

Mohammed El-Adawy [13] investigated the effect of ZnO nanoparticles. He incorporated ZnO with biodiesel-diesel blends. It has been found out that the combustion improved by ZnO nanoparticles and increasing the BTE by minimizing heat losses. The objective was also supported by Rajak et al. [14] who observed that the incorporation of ZnO nanoparticles into biodiesel blends led to higher BTE than the simple biodiesel.The contribution of CuO nanoparticles in enhancing BTE has also been discussed in the literature. Harish et al. [15] showed that the CuO nanoparticles increase the combustion efficiency of biodiesel blends resulting in higher BTE. Akroot et al. [16] concluded that the thermal efficiency of biodiesel-diesel fuel mixtures was enhanced through the inclusion of CuO nanoparticles. Siddiqui et al. [17] have reviewed that Fe_2_O_3_ nanoparticles are capable of improving BTE. According to the study, Fe_2_O_3_ nanoparticles facilitated combustion and produced high thermal efficiency. Thiagarajan et al. [18] stated that incorporating the biodiesel blends with Fe_2_O_3_ nanoparticles enhanced the BTE. Studies have also been made on the effects of manganese oxide (MnO_2_) nanoparticles on BTE. Senthil et al. [19] showed that, MnO_2_ nanoparticles enhanced the combustion process thus increasing the BTE in biodiesel-diesel blends. It was also found by Rajpoot et al. [20] that the existence of MnO_2_ nanoparticles promoted the thermal stability of biodiesel blends. Reddy et al. [21] conducted another study with focus on the effects of silicon dioxide (SiO_2_) nanoparticles on BTE. The findings obtained from the study showed that the addition of SiO_2_ nanoparticles led to increased BTE because of enhanced combustion efficiency and minimal heat losses. Ghanati et al. [22] also found that SiO_2_ nanoparticles increased the thermal stability of biodiesel blends. Rajendran et al. [23] observed higher value of BSFC for biodiesel blends in comparison to that for pure diesel as it has lower calorific value. But the study also pointed out that, although the value of BSFC was increased, the benefit of biodiesel was more useful due to its association with the environment. Hasnain et al. [24] revealed that, although the BSFC of biodiesel blends was slightly higher but the overall biodiesel fuel savings were significant considering its renewable nature. Apart from metal oxide nanoparticles, there have been attempts to use hybrid nanoparticles. BTE has been studied under the influence of hybrid nanoparticles [25]. The results showed that hybrid nanoparticles greatly increase BTE through improvement of combustion processes and minimization of heat losses [26,27]. It was confirmed by Anbarsooz [28] who pointed out that the use of hybrid nanoparticles enhanced the thermal stability of biodiesel/ diesel emulsion. Incorporation of Metal oxide nanoparticles appear to have high possibilities of decreasing the BSFC [29,30]. Mohapatra et al. [31] attempted to study the effect of Al_2_O_3_ nanoparticles on biodiesel-diesel blends. The findings showed the enhancement of combustion efficiency by utilizing Al_2_O_3_ nanoparticles with biodiesel blends had led to the reduction of BSFC. The investigation of literature shows that metal oxide nanoparticles can improve combustion characteristics, but the magnitude of the improvement highly depends upon the oxide bonds, dispersion method and loading procedure. Cerium oxide (CeO_2_) and Titanium oxide (TiO_2_) have widely been reported to minimize the ignition delay and improve the heat release rate at peak of engine operation.[2]. Iron and Manganese Oxides provide effective catalytic sites that can promote complete oxidation of the intermediate species. On the other hand, silicon di oxide improves dispersion stability and fuel handling properties. Across all studies, the benefits are bound by the practical limits such as the dosage of the nano particles, agglomeration of nanoparticles, deposit formation risk in engine and spray characteristics of NPs. This comparative picture motivates the use of the nanoparticle with high surface area, high thermal stability, environmental benefit, catalytic activity and low-cost characteristics. MgO bears all these characteristics and appears as a promising selection for the present study.[32,33].

Recent studies have shown that the incorporation of metal and metal oxide nanoparticles into biodiesel–diesel blends can significantly enhance combustion, atomization, and thermal conversion efficiency in compression ignition (CI) engines. Mangesha et al. [34] reported that dual nano-additives in cottonseed biodiesel improved brake thermal efficiency (BTE) and reduced emissions, demonstrating a synergistic catalytic effect. Kumar et al. [35] found that TiO₂ nanoparticles combined with hydrogen enrichment in *Madhuca* biodiesel enhanced ignition delay and combustion stability, leading to increased efficiency. Similarly, Jayabal [36] observed that hybrid nanoparticles with hydrogen addition in algae biodiesel–diesel blends reduced carbon monoxide (CO) and unburned hydrocarbon (HC) emissions through improved oxidation. Janaki et al. [37] confirmed that magnesium oxide (MgO) nanoparticles and hydrogen enrichment improved the performance of Mahua oil biodiesel, achieving lower brake-specific fuel consumption (BSFC) and higher BTE.

Other investigations have further established the role of nanoparticles in optimizing engine operation. Vellaiyan [38] demonstrated that algae biodiesel blended with nanoparticles, treated using an electrostatic precipitator, achieved enhanced combustion performance and reduced nanoparticle emissions. Nathamuni et al. [39] also reported higher in-cylinder pressure and faster heat release rates in biodiesel–nanoparticle blends, indicating better combustion propagation. Çılgın [40] explored the combined use of single-walled carbon nanotubes (SWCNT) and MgO nanoparticles, revealing simultaneous improvements in BTE and emission characteristics. Similarly, Demir et al. [41] and Hassan et al. [42] found that silver and other metallic nanoparticles synthesized through green routes improved oxidation rates and lowered CO and NOₓ emissions when blended with biodiesel derived from waste oils. Ismael et al. [43] further demonstrated that mono and hybrid nanoparticles in water-in-biodiesel emulsions enhanced thermal efficiency and minimized soot formation. Collectively, these studies establish that nanoparticle-assisted biodiesel combustion offers a viable approach to improving fuel economy, emission performance, and overall sustainability in CI engine applications.

Table 1 briefly provides the NPs influence on performance metrics of diesel engines.

**Table 1:** Comparison of the Biodiesel blends with Metal oxide particles on the performance parameters of CI engine.

Name of the Paper	Engine Used	Fuel Used	Additive Used	Quantity	BTE	BSFC
Venu and Appavu	Single-cylinder diesel engine	Polanga biodiesel	Al_2_O_3_	25 ppm	↑	↓
Venu et al.	Single-cylinder DI diesel engine	Jatropha biodiesel (20%) + ethanol (10%) + diesel (70%)	Al_2_O_3_	10 ppm	↓	↑
Mohan and Dinesha	4-stroke, single-cylinder, water-cooled, DI diesel engine	Waste cooking oil biodiesel (20%) + H2O2 (1.5%) + diesel (78.5%)	CeO_2_	40 ppm	↑	↓
Alex et al.	4-stroke, single-cylinder, air-cooled, DI diesel engine	Diesel (100%)	CeO_2_	15 ppm	↑	↑
Ağbulut et al.	Naturally aspirated, air-cooled, DI diesel engine	Diesel (100%)	CuO	1000 ppm	↑	↓
Sathish et al.	Single-cylinder, 4-stroke diesel engine	Waste cooking oil biodiesel (20%) + diesel (80%)	CNT	100 ppm	↑	↑
Venu and Madhavan	Four-stroke, air-cooled, single-cylinder DI diesel engine	Diesel (70%) + biodiesel (20%) + ethanol (10%)	Al_2_O_3_	25 ppm	–	↓
Al-Kheraif et al.	Air-cooled, single-cylinder, four-stroke CI engine	Candle nut and soap nut biodiesel (20%) + diesel (80%)	Al_2_O_3_	25 ppm	↑	↑
Fayad and Dhahad	DI diesel engine, water-cooled, four-cylinders	Butanol (20%) + diesel (80%)	Al_2_O_3_	30 mg/L	↑	↓
Manigandan et al.	Single-cylinder, 4-stroke, CI engine with dual combustion mode	Hydrogen (0.2 kg) + diesel (0.78 kg)	TiO_2_	0.02 kg	↑	↓
Gad et al.	Single-cylinder, air-cooled, 4-stroke diesel engine	Waste cooking oil biodiesel (20%) + diesel (80%)	TiO_2_	25 mg/L	↓	↑

Complex experimentation requires significant finance and time to obtain the best operating conditions for an engine [44]. Statistical approaches along with numerical methodologies have emerged as a viable solution to these problems. Several optimization methods like Artificial Neural Networking (ANN), Taguchi, Response Surface Methodology (RSM) can be applied to calculate the optimal operating parameters while reducing the need of large number of experiments [45]. A significant advantage of the RSM over ANN is its ability to optimize using hill-climbing technique. RSM also uses simpler structure for the optimization that reflects in lower computational costs. RSM was reported to provide better estimation results than the ANN for limited number of the experiments. Khan et al. [46] performed experimentation on biodiesel-hydrogen engines with cerium oxide particles. They utilized RSM for the optimization of the engine performance. Shirneshan et al. performed the experimentation on the biodiesel-ethanol mixtures at different engine loads and speeds with RSM. They found regression coefficient values between 0.945 and 0.993 [47].

Table 2 compared the optimization approaches for the RSM based optimized studies. While those studies optimized both performance and emission outputs through RSM alone. However, the current work utilizes hybrid Taguchi screening and RSM validation strategy enabling the resource efficient performance optimization.

**Table 2:** Comparison of the RSM approach for the optimized studies.

Study	Focus & Fuel Blend	Optimization Methodology	Main Variables	Key Performance Metrics	Distinctive Insights
Current Study (Taguchi + RSM, MgO-biodiesel)	Biodiesel + MgO nanoparticle blends	Taguchi L18 orthogonal → RSM confirmatory	Biodiesel (%), Engine Speed, Load, MgO (g)	BTE, BSFC	Focused on energetic efficiency; efficient screening via Taguchi, validated with RSM
Engine performance using hydrogen diesel blends [48]	Cottonseed biodiesel + H₂ + EGR	RSM optimization	CSB fraction, Hydrogen %, EGR rate	Performance & emissions	Emphasizes H₂ enrichment and NOₓ control via EGR
Biodiesel, hydrous hydrazine and nano catalysts based engine performance [49]	Biodiesel + hydrous hydrazine (H₂ carrier) + CuO nanocatalysts	Likely RSM or similar	Biodiesel %, Hydrazine %, Nano catalyst loading	Combustion & emission metrics	Uses hydrazine as H₂ source + catalytic enhancement
Optimization of Spirulina biodiesel-ammonium hydroxide blends with EGR for diesel engine [50]	Spirulina biodiesel + NH₄OH + EGR	RSM for multi-parameter optimization	MES %, AH %, EGR %	Performance & emissions	Integrates biofuel from Spirulina and hydrogen-rich AH with emissions control

The novelty in this study lies in both selection of additives and methodological framework. While broad literature exists on metal oxide nanoparticles including CeO₂, TiO₂, ZnO, and CuO for biodiesel enhancement. MgO utilization remains underexplored as compared to above mentioned nanoparticles despite various advantages. This study addresses this gap by applying the L_18_ Taguchi orthogonal array and quantifying the operating conditions through the RSM proving robust approach for performance enhancement. The use of SFR along with Taguchi adds the robustness in the model and reduces the high experimental costs associated with full scale studies. Together, this dual novelty (MgO nanoparticle blends and Taguchi – RSM) differentiates this work from the existing literature and provides a new framework for sustainable diesel engine performance enhancement.

## 2. Materials and methodology

### 2.1. Experimental methodology

**Fig 1:**
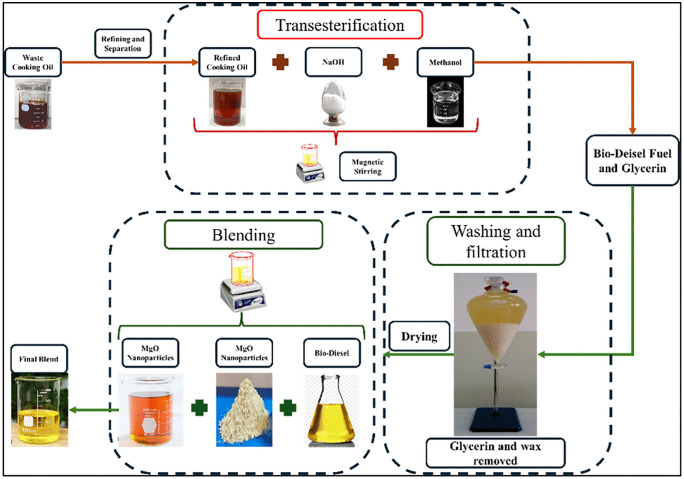
Schematic of Production of the Biodiesel Methanol was added with the molar ratio of six to one. The 47grams of the NaOH dosage was used for every 5 kg of the WCO which is in accordance with ASTM D6751. It was continuously stirred at the speed of 250 RPM for an hour at a designated temperature range. Finally, the products of the reaction were separated by means of measuring flask and the final product was then heated up with temperature of 100°C for the purification and the obtained biodiesel was tested according to ASTM standards. The recorded properties are shown in Table 3.

Fig 1 describes the schematic of the production of the Biodiesel using transesterification process and mixing with the MgO nanoparticles. Biodiesel was obtained from waste cooking oil through transesterification with methanol in the presence of catalyst. First the filtration and purification of biodiesel were performed, and the insoluble particles were removed from the oil. For this purpose, a filter cloth of 2 mm thickness was utilized, and the purified oil was placed in a transesterification reactor operating at the temperature range of 50–60°C.

**Table 3:** Characteristics of the Biodiesel utilized for experimentation.

Characteristic	Specification
**Flash Point**	189.5
**Calorific Value (kJ/kg)**	39125
**Cetane Number**	49
**Density**	0.88

The equipment used for testing purposes includes GEW 80/DWE-60/EWS-150-L water cooled, 4-cylinder commercial diesel test engine. A dynamometer with maximum absorbing power of 150 horsepower was coupled with the engine controlling the speed and torque.

**Fig 2:**
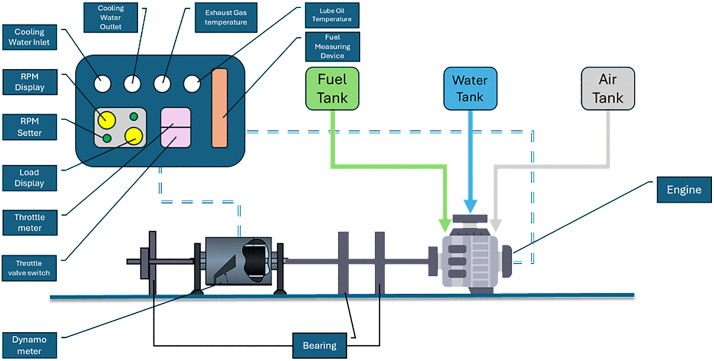
Experimental Setup Schematic.

Fig 2 shows the experimental setup and the specifications of the test engine and dynamometer are shown in Table 4.

**Table 4:** Test Bed engine Specifications.

Characteristics	Specifications
**Engine Manufacturer**	TD 23
**Bore (mm)**	89
**Stroke (mm)**	92
**Cylinder volume (L)**	2.289
**Number of Cylinders**	04
**Peak Torque (N.m.)**	134.35 at 2000 RPM
**Power (kW)**	44.13
**Dynamometer model**	EWS 150 L
**Dynamometer Absorbing Power (K W)**	110
**Compression Ratio**	21.9

The engine was coupled to an electrically controlled dynamometer, which means the coupling and loaded conditions are applied using the knob from the control panel. The engine was first allowed to run for 10 minutes to achieve stability and then speed was incrementally increased to desired RPM. This setup requires manual measurement of fuel consumption therefore needed a stopwatch to record count of fuel consumption in specific time.

**Fig 3:**
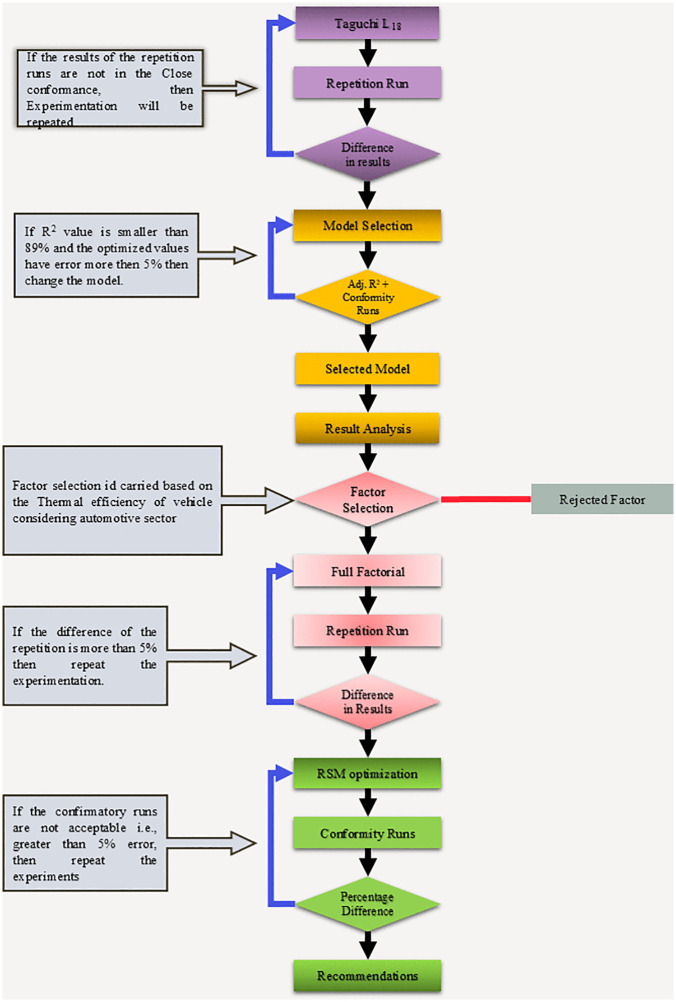
Flow chart for the Experimentation and Optimization. This study focuses on the energetic performance of MgO – doped biodiesel – diesel blends, using BTE and BSFC as primary variables. These two-performance metrics were selected for initial optimized screening because they directly quantify the nest useful energy output and fuel economy, enabling efficient identification of promising fuel/additive combinations.

The specific fuel consumption and thermal efficiency were recorded at different combinations of Engine speed, biodiesel blends, engine load and magnesium oxide particles which are shown in Table 5. The results were recorded at the engine speed of 1200,1600 and, 2000 RPM along with the varying load at 25%, 50% and 75%. The MgO was added at 0,0.02 and 0.04 grams. There are total of six fuel levels with level 1 at 0% biodiesel and level 6 at 20% biodiesel. The biodiesels were level at BD0, BD4, BD8, BD12, BD16 and BD20. Magnesium oxide was mixed with fuel using magnetic stirrers so that proper mixing is ensured. Fig 3 provides the Flowchart of the Experimentation and Optimization methodology.

**Table 5:** Selected L_18_ special array experimentation design.

Experiment Number	A Fuel Biodiesel %	B Speed RPM	C Load %	D MgO g
1	0	1200	25	0
2	0	1600	50	0.02
3	0	2000	75	0.04
4	4	1200	25	0.02
5	4	1600	50	0.04
6	4	2000	75	0
7	8	1200	50	0
8	8	1600	75	0.02
9	8	2000	25	0.04
10	12	1200	75	0.04
11	12	1600	25	0
12	12	2000	50	0.02
13	16	1200	50	0.04
14	16	1600	75	0
15	16	2000	25	0.02
16	20	1200	75	0.02
17	20	1600	25	0.04
18	20	2000	50	0

### 2.2. Design of experiments

Design of Experiment is important for financial balancing and optimization of the experimental process to reduce expenses and time [51]. Therefore, the Taguchi experimental design is selected for obtaining critical scenarios and points in the experimental design. A specific orthogonal array of L_18_ is utilized for experimentation. In this work, biodiesel blends were prepared at 4–20 vol%. The selected blends align with the international fuel policies that mandate biodiesel content in range of B4 – B20. Meng et. al. [52] concluded that biodiesel blend at 30% caused increase in viscosity and decreasing the combustion properties. Also, blending percentage exceeding more than 30% showed poor spray atomization, injector choking and reduced volatility [53]. Hence, the range of 4–20% was chosen to cover both low and moderate level of biodiesel addition with regulatory limits [54,55]. The MgO concentration range was derived from the dispersion and combustion stability studies [56–58]. Several studies showed that range between 40–50 mg provides optimal dispersion and catalytic activity. While values under 10 mg provide negligible effect and values over 50 mg provide fuel instability and agglomeration [59]. The experimental plan was constructed using the mixed level Taguchi L18 orthogonal array to efficiently handle factors with varying levels. Six orthogonally balanced combinations were selected to match the required biodiesel levels while maintaining orthogonal relationships with the factors. Engine speed (1200, 1600, 2000 RPM), Load (25%, 50% 75%), and MgO dosage (0, 0.02, 0.04) each had three levels and were assigned directly to single columns. Table 5 represents the selected L_18_ special array experimentation design.

The process flowchart of the experimentation is summarized in Fig 3. The results obtained from the L_18_ were evaluated. Repetition runs were performed to check the validity of the experimental study. Once the results were obtained, different fitting models were used to analyze the feasibility of the design. The fitting models included general linear models (GLM), Linear regression models (LR) and stepwise forward regression (SFR). The models having less than 89% adjusted R-Squared values were eliminated and models with poor prediction for confirmatory runs against parameter settings were also eliminated. Runs were performed for the Brake specific fuel consumption (BSFC) and Brake Thermal Efficiency (BTE).

### 2.3. Uncertainty analysis

An uncertainty analysis was conducted for the evaluation of the reliability and precision of the experimental measurements. The root mean squared (RMS) method and standard deviation of the mean were used to assess the uncertainties associated with performance parameters, the formula utilized for the uncertainty was following [60]


$$U_{x}=\sqrt{\frac{\text{1}}{N^{\prime }}\sum_{i=\text{1}}^{N^{\prime }}{\left(X_{i}-\overset{―}{X}\right)}^{\text{2}}}$$ (1)

Where

• $U_{x}$ = uncertainty of the measured value

• $X_{i}$ = observed value from each trial

• $\overset{―}{X}$ = average of observed values

• N′ = number of trials

Overall uncertainty was calculated from multiple parameters using [61]:


$$\;U_{total}=\sqrt{\sum {\left(\frac{\partial R}{\partial x_{i}}\cdot U_{x_{i}}\right)}^{\text{2}}}$$ (2)

Where:

• R = the result of the output

• $x_{i}$ = each individual input

• $\frac{\partial R}{\partial x_{i}}$ = partial derivatives of the output with respect to input

Table 6 shows the uncertainty of the experiment.

**Table 6:** Uncertainty of the experiment.

Parameter	Range	Accuracy	Uncertainty (%)
**Fuel Volume (V)**	0–500 mL (burette)	±0.2 mL	±0.04%
**Time (t)**	0–300 sec (stopwatch)	±1 sec	±0.50%
**Speed (RPM)**	0–3600 RPM	±10 RPM	±0.28%
**Torque (Nm)**	0–100 Nm	±0.8 Nm	±0.80%


$$U_{total}=\sqrt{(\text{0}.\text{0}\text{4}{)}^{\text{2}}+(\text{0}.\text{5}{)}^{\text{2}}+(\text{0}.\text{2}\text{8}{)}^{\text{2}}+(\text{0}.\text{8}{)}^{\text{2}}=\;\pm \text{0}.\text{98}\%}$$ (3)

The total uncertainty was 0.98%, suggesting that the model was accurate and reliable. This value accounts for all the critical parameters influencing BTE and BSFC thus confirming robustness and reproducibility of the results.

### 2.4. MgO selection

MgO nanoparticles were selected owing to their strong catalytic oxidation potential, high surface area and thermal stability. Moreover, it is inexpensive compared to other nanoparticles. The Table 7 provides a comparison of the metal oxide nanoparticles for the biodiesel combustion enhancement.

**Table 7:** MgO selection.

Property	MgO	TiO₂	CeO₂	ZnO	Justification for MgO
**Surface Area (m²/g)**	180–250	50–100	60–110	90–130	High surface area promotes better dispersion and catalytic activity
**Thermal Stability (°C)**	>2800	~1800	~2400	~1975	MgO resists thermal decomposition under high combustion temperatures
**Oxygen Storage/Release Capacity**	Moderate (acts as a base donor)	Low	High (Ce⁴⁺ ↔ Ce³ ⁺ cycling)	Moderate	Sufficient for biodiesel, without reactive oxygen surges that may induce NOx formation
**Catalytic Activity (Reaction Sites)**	High (basic sites)	Moderate (photocatalytic)	High (redox potential)	Moderate	MgO supports ester bond oxidation and combustion improvement
**Toxicity/Environmental Impact**	Non-toxic, eco-friendly	Benign	Low–Moderate toxicity	Slightly toxic	Safe handling, no regulatory restrictions
**Relative Cost (per kg, USD)**	10–15	40–60	80–100	35–50	MgO is low-cost and readily available
**Synergy with WCO Biodiesel**	High (proven in several studies)	Moderate (less reported)	High	Moderate	MgO shown to reduce BSFC and improve BTE with WCO blends

### 2.5. Biodiesel characterization

**Fig 4:**
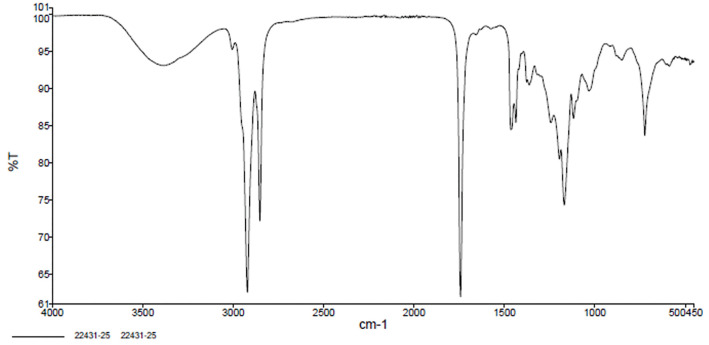
FTIR Analysis of the Biodiesel. GC analysis of the biodiesel was performed using FID detector. This technique was utilized in literature to find the major components in the biodiesel [63]. The analysis showed that major components including methyl palmitate, menthol oleate and methyl linoleate, which are commonly found in biodiesel derived from the vegetable oils. The results affirm the successful conversion of the triglycerides to biodiesel. These findings align with the studies performed by Singh et. al. [64] suggesting a standard Fatty acid methyl esters (FAME) composition across different biodiesel types. The Table 8 provides the results of the GC analysis.

The FTIR spectrum exhibited a strong peak at 1442 cm^-1^ corresponding to C = O stretching of ester groups. Further peaks at 2922 cm^-1^ provides the CH_2_ stretching, 2853 cm^-1^ provides symmetric CH_2_ while the 1170 cm^-1^ provides C – O stretching. Fig 4 shows the FTIR spectrum of the biodiesel [62].

**Table 8:** GC – FID of the biodiesel.

Parameter	Unit	Results
Methyl laurate (C12:0)	%	0.55
Methyl Tri decanoate (C13:0)	%	0.19
Methyl myristate (C14:0)	%	1.47
Methyl pentadecanoate (C15:0)	%	0.11
Methyl palmitate (C16:0)	%	62.15
Methyl stearate (C18:0)	%	3.38
Methyl heneicosanoate (C21:0)	%	0.78
Methyl eleiate (C18:1)	%	21.44
Methyl linoleate (C18:2)	%	5.44
Methyl gamma-linoleate (C18:3)	%	1.28
Methyl docosadienate (C22:2)	%	2.39
Myristoleic acid (C14:1)	%	0.10
Methyl linoleate (C18:3) (ALA)	%	0.73

After the systematic analysis, the factors and their levels were selected for performance evaluation using full factorial design. After this RSM optimizations were validated by performance confirmatory runs on predicted values. The recommendations are made based on fully validated RSM optimization.

## 3. Results and discussion

### 3.1. Repetition analysis and orthogonal array – L_18_

The results of the selected Design of Experiments (DOE) were obtained by performing the experiments in random order to avoid the biasness in the results. Table 9 shows the BTE and BSFC values for the experimental combination based on Table 5.

**Table 9:** BTE and BSFC values for the experimental combination.

Experiment Number	A	B	C	D	BSFC g/kW	BTE %
	Speed	Load	Fuel	MgO		
	RPM	%	Biodiesel %	g		
1	1200	25	0	0	319.00	18.50
2	1600	50	0	0.02	327.45	20.54
3	2000	75	0	0.04	332.61	21.48
4	1200	25	4	0.02	317.00	20.80
5	1600	50	4	0.04	316.00	20.94
6	2000	75	4	0	329.77	21.07
7	1200	50	8	0	320.94	21.24
8	1600	75	8	0.02	323.45	22.17
9	2000	25	8	0.04	313.00	19.20
10	1200	75	12	0.04	318.06	23.27
11	1600	25	12	0	313.00	19.40
12	2000	50	12	0.02	340.00	20.24
13	1200	50	16	0.04	324.00	22.04
14	1600	75	16	0	342.47	22.17
15	2000	25	16	0.02	304.00	18.70
16	1200	75	20	0.02	310.00	23.27
17	1600	25	20	0.04	315.00	20.00
18	2000	50	20	0	350.00	18.30

The maximum and minimum BTE values were obtained in **Experiment 10 and Experiment 16.** A thorough evaluation of the results shows as follows:

- To get the best BTE and BSFC conditions, there must be a tradeoff between both BTE and BSFC.
- Critical input parameter interactions are important to select the optimal conditions.

**Fig 5:**
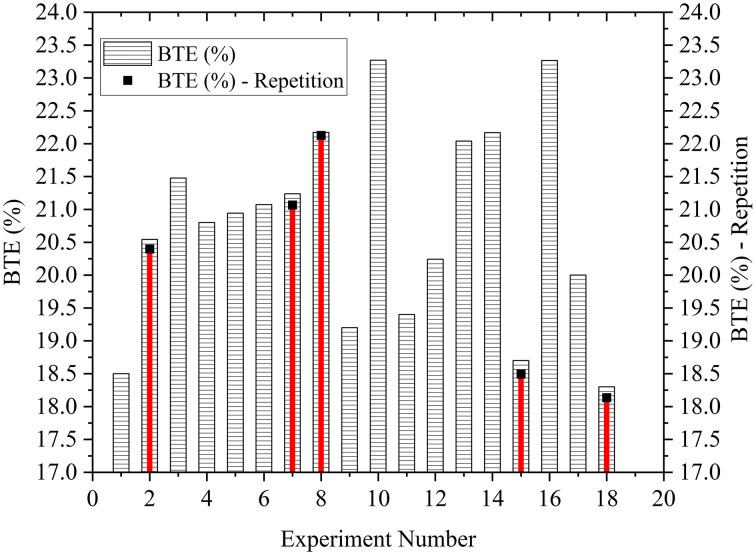
Repeatability analysis in the BTE.

**Fig 6:**
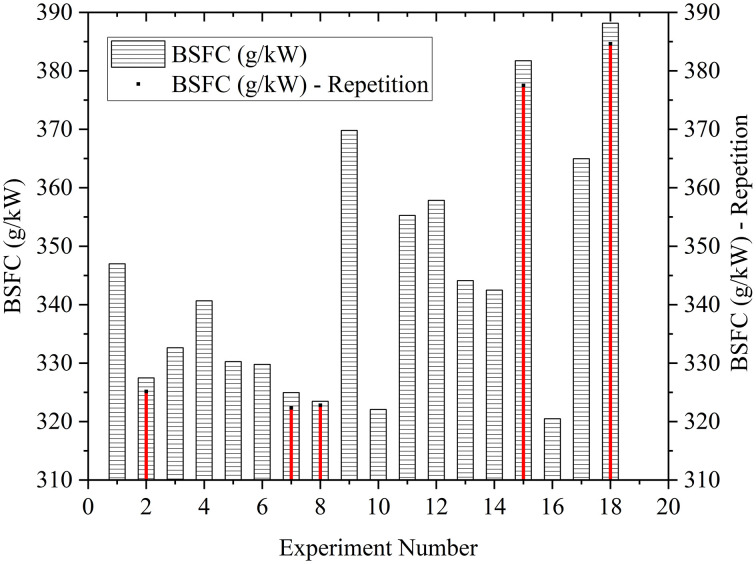
Repeatability analysis in the BSFC.

The reliability of the experiment is guaranteed by repeating the selected points. The experiments with minimum and maximum BTE and BSFC were selected for repetition. Figs 5 and 6 provide the insights into the repeatability analysis in the experimental data. Thus, showing confidence in the feasibility and accuracy of the experiment.

### 3.2. SFR for the BTE

BTE was analyzed in Minitab Statistical Software 22 using GLM, LR and SFR. GLM and LR could not qualify the criteria used for further analysis. The elimination of the GLM and LR models was based on three statistical criteria. First, adjusted R^2^ values below 89% were considered insufficient. Second, confirmatory runs were performed and values having more than 5% error were considered unacceptable. Third, GLM and LR indicated poor handling of higher order interactions while SFR remains consistent. These criteria provided both accuracy and robustness in the predictive capability. SFR was selected as it can also provide insights into the intricate relationships between the input parameters. The R-Squared and Adjusted R-Squared values for GLM and LR are shown in Table 10.

**Table 10:** The R-Squared and Adjusted R-Squared values for GLM, LR and SFR.

Sr. No.	Regression Models	BTE			
–	–	R^2	R^2 (Adj.)	∆R^2	∆R^2(Adj.)
A	GLM	95.51	87.29	–	–
B	LM	89.66	86.48	–	–
C	SFR	–	–	–	–
1	Fuel, Load, RPM, MgO	89.66	86.48	0	0
2	Fuel, Load, RPM, MgO, Fuel*Fuel	92.69	89.64	3.03	3.16
3	Fuel, Load, RPM, MgO, Load*Load	90.48	86.52	−2.21	−3.12
4	Fuel, Load, RPM, MgO, RPM*RPM	90.05	85.9	−0.43	−0.62
5	Fuel, Load, RPM, MgO, MgO*MgO	90.7	86.82	0.65	0.92
6	Fuel, Load, RPM, MgO, Fuel*Load	89.8	85.55	−0.9	−1.27
7	Fuel, Load, RPM, MgO, Fuel*RPM	92.48	89.35	2.68	3.8
8	Fuel, Load, RPM, MgO, Fuel*MgO	89.7	85.41	−2.78	−3.94
9	Fuel, Load, RPM, MgO, Load*RPM	89.74	85.46	0.04	0.05
10	Fuel, Load, RPM, MgO, Load*MgO	91.19	87.52	1.45	2.06
11	Fuel, Load, RPM, MgO, RPM*MgO	89.75	85.48	−1.44	−2.04

In SFR, the main factors were kept in every model while the interacting factors were changed to identify the relationship between inputs and outputs. R-squared and Adjusted R-squared were measured for each case and tabulated in Table 10. R-square alone can lead to wrong interpretation because it can increase by adding terms. Also, the term with no actual effect can also cause an increase in R-squared. Thus, it is necessary to observe the model considering the adjusted R-squared value. Such a problem can be seen in Table 10. The first case included only the main factors. In cases 2–11, high order interacting factors were added to identify the factors affecting the results.

The statistical adequacy of Taguchi models for BTE was evaluated using coefficient of determination R^2,^ adjusted R^2^. For the selected Model of BTE in SFR was 92.48% R^2^ and 89.5% adjusted R^2^ indicating excellent agreement in the predicted and experimental data. The predicted equation for the selected model of the BTE is given below


BTE=18.75 + 0.276 Fuel − 0.000471 Speed + 0.04560 Load + 16.21 MgO − 0.000164 Fuel*Speed  (4)

The Taguchi method utilizes the concept of the signal to noise ratio (S/N) to measure the quality characteristics from desired value. The S/N ratio formulae were:


$$\text{Larger the better: }S/N\;=\;-\text{1}\text{0}{log}_{\text{1}\text{0}}\;(\frac{\text{1}}{n}\;\sum_{i=\text{1}}^{n}\frac{\text{1}}{y_{i}^{\text{2}}})$$ (5)

$$\text{Smaller the better: }S/N\;=\;-\text{1}\text{0}{log}_{\text{1}\text{0}}\;(\frac{\text{1}}{n}\;\sum_{i=\text{1}}^{n}y_{i}^{\text{2}})$$ (6)

Where $y_{i}$ represents the measured output for the $i^{th}$ trial and n is the number of repetitions. The probability assumption is that the experimental errors follow a normal distribution and factor effects are additive. This approach reduces noise and provides the robustness against noise levels. The optimum factor levels obtained from the Taguchi analysis were validated by performing three repeated experimental trials at the optimal settings. The average of these predicted values was compared with the values from the Taguchi model. Validation was considered successful when the deviation between the predicted and experimental values was less than 5%.

**Fig 7:**
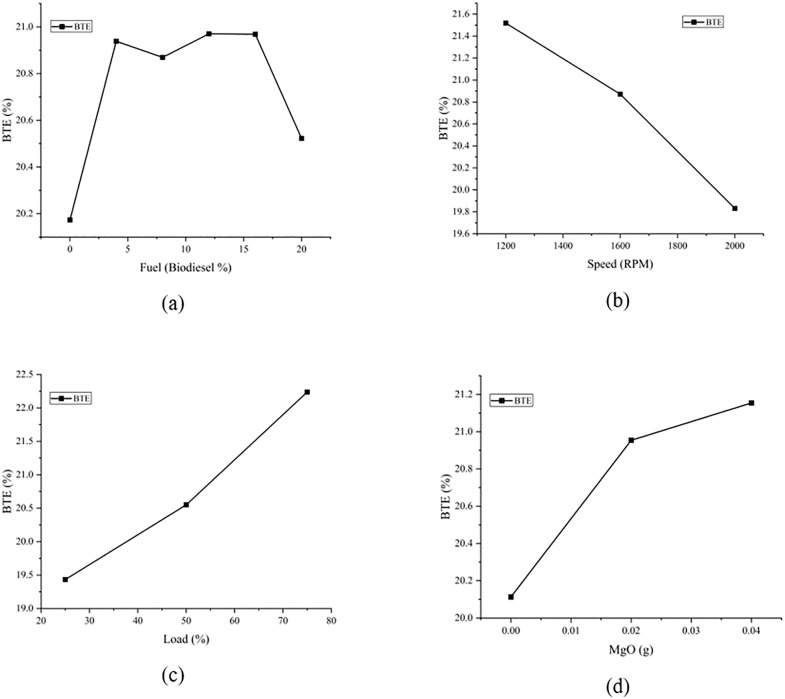
Main effects Plots of the BTE with (a) Biodiesel percentage (b) Speed (c) Load percentage (d) MgO Content.

The effects of the main plots are shown in Fig 7. BTE is plotted on the ordinate while Biodiesel percentage in fuel, load on the engine, MgO concentration in the fuel and speed of the engine are on the abscissa. The maximum BTE was obtained on the 12% biodiesel blend. The biodiesel blend with diesel can increase the BTE, therefore it can be seen that the lowest BTE is at 0% biodiesel blend. Another trend can be seen that by increasing the Biodiesel more than 16% there is decrease in the BTE. Biodiesel has lower calorific value causing the overall energy content of the mixture to decrease thus reduction in BTE. Higher biodiesel blends can affect the atomization and have high viscosity, thus causing the decrease in the BTE. Biodiesel has higher ignition delay and low peak flame temperatures causing incomplete combustion and lower BTE [65].

BTE has an inverse relation with speed. It is because the increase in speed can cause incomplete combustion thus decreasing the BTE. At higher speeds, the friction in the engine components can increase which causes high percentage of the fuel being utilized, the heat causing decrease in the BTE. At higher speeds, the chance of incomplete combustion can increase causing the low energy extraction from the fuel leading to the low BTE [65]. With the increase in the engine speed, the rate of the cooling of the engine system also decreases thus resulting in energy losses and causing decrease in BTE. The pumping losses in the engine can also increase causing the reduction in the BTE.

BTE is increasing with the increase in the load percentage. At higher loads, the rate of combustion is increased as more fuel is injected leading to higher BTE. With the higher loads, the proportion of the energy lost as heat increases is relative to the energy converted to useful work. This is due to the better combustion and thermal efficiency at higher loads. At higher loads the frictional loads remain constant, so at higher loads, these losses constitute a smaller fraction of the total power output [66].

BTE was increased with the MgO concentration because the oxygen content in nanoparticles improve combustion efficiency. Better combustion means better fuel utilization thus increasing the BTE. Also, the catalytic nature of the MgO can also provide the promotion in the complete combustion. Thus, we can see that the fuel with no MgO has less BTE as compared to the fuel with the MgO.

Table 11 is representing the optimal input values selection from the factor plots and the theoretical values were determined along with the experimental values, and the error was less than **5%.**

**Table 11:** Optimal value selection for BTE.

Maximize	Fuel	Speed	Load	MgO	BTE Theoretical	BTE Experimental	% Error
BTE	12	1200	75	0.04	23.5342	23.27	1.12%

**Fig 8:**
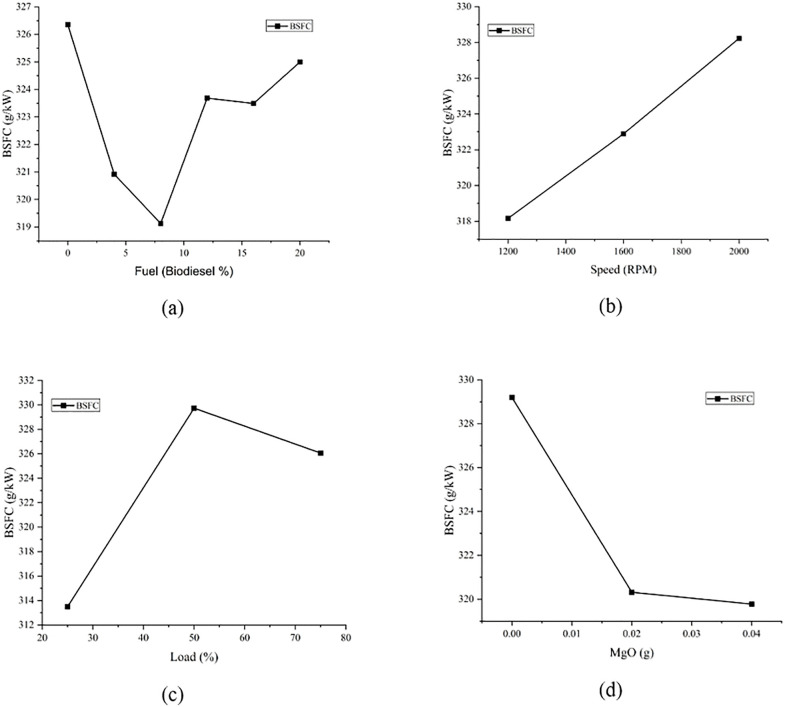
Main effects Plots of the BSFC with (a) Biodiesel percentage (b) Speed (c) Load percentage (d) MgO Content.

The subsequent decrease in the BSFC is due to the improved combustion due to high oxygen content in biodiesel. Also, the lubricating properties of biodiesel are better than conventional diesel thus decreasing the internal engine friction. Fig 8 shows the main effect plots for the BSFC. At higher loads, the combustion efficiency can reach a point where an increase in the fuel does not correspond to the increase in the power output [67]. The engine generated more heat at higher loads causing high thermal losses which increased the BSFC. With increased MgO concentration, the BSFC decreases because MgO acts as the catalyst during the combustion causing complete combustion to occur in the engine thus decreasing BSFC. MgO particles have high thermal conductivity, which provides better heat distribution in the combustion chamber by reducing hotspots and decreasing BSFC. The optimal values of input for BSFC were obtained in Table 12.

**Table 12:** Optimal value selection for BSFC.

Minimize	Fuel	Speed	Load	MgO	BSFC Theoretical	BSFC Experimental	% Error
BSFC	8	1200	25	0.04	306.983	292.861782	4.60%

The optimum factor levels obtained from the Taguchi analysis were validated by performing three repeated experimental trials at the optimal settings. The average of these predicted values was compared with the values from the Taguchi model. The experimental values fell within the 95% confidence interval of prediction values.

### 3.3. DOE Factor Selection

The biodiesel mixture of DB12 and DB20 predicted optimized performance for BTE and BSFC, respectively. However, the mixture with the 0.04g of MgO resulted in optimization of both parameters. The load and the speed demonstrated linear trend for both output parameters. From an economic standpoint, a tradeoff can be made between BTE and BSFC as the higher BTE will provide better performance in the automotive industry and heavy-duty industry [68]. Therefore, the primary criteria for parametric selection for design of experiment (DOE) will be BTE.

A three-level full factorial design is created using 0.04g of MgO for optimization while keeping the load constant at 75%. The effect plot demonstrates a swift decline in the BTE over the rise in the biodiesel value in the fuel mixture in Fig 7. Nonlinearity can be observed in the speed values as well. Therefore, to determine the impact of these variations, the values of Biodiesel as BD12, BD16 and BD20 were selected to monitor the sudden change. The speed levels were taken as 600 rpm, 800 rpm and 1000 rpm to determine its extended impact. Secondly, this can also improve operating points at lower speeds of operations. Table 13 demonstrates the full factorial design with complete results.

**Table 13:** Three-level full factorial design for RSM for heavy duty Industry.

Case	RPM	Load	Fuel	MgO	BSFC	BTE
1	600	75	8	0.04	309.56103	24.71
2	600	75	12	0.04	310.016718	24.69
3	600	75	16	0.04	309.872406	24.8
4	800	75	8	0.04	313.371998	24.29
5	800	75	12	0.04	311.827686	24.29
6	800	75	16	0.04	312.383374	24.21
7	1000	75	8	0.04	315.432966	23.8
8	1000	75	12	0.04	315.358654	23.9
9	1000	75	16	0.04	314.494342	23.95

The best efficiency was obtained in experiment one while the least BSFC was found in experiment four. These results suggest that the highest efficiency can be obtained at lower speeds while the BSFC can be reduced at higher biodiesel composition in the fuel.

Several studies show that the cars cruise on the speeds between 1000 and 3000 RPM [69,70]. For the automotive industry, the speed values were changed between 1200 RPM and 2000 RPM, and these values were also in conformity with the factorial graphs. Table 14 shows the three-level full factorial design for RSM for automotive industry.

**Table 14:** Full factorial design for RSM for Automotive Industry.

Case	Speed	Fuel	Load	MgO	BSFC	BTE
1	1000	75	8	0.04	314.582966	23.5
2	1000	75	12	0.04	315.038654	23.8532
3	1000	75	16	0.04	314.894342	23.8
4	1200	75	8	0.04	318.393934	23.24
5	1200	75	12	0.04	316.849622	23.39
6	1200	75	16	0.04	317.40531	23.35
7	1400	75	8	0.04	320.454902	23.01
8	1400	75	12	0.04	320.38059	23.01
9	1400	75	16	0.04	319.516278	23.04

The maximum BTE and minimum BSFC were in case 2 and case 1, respectively. The results suggest that at higher speed, the BSFC will be higher and BTE will be lower [71,72].

### 3.4. Response Surface Methodology (RSM)

#### 3.4.1. Heavy duty industry.

**Fig 9:**
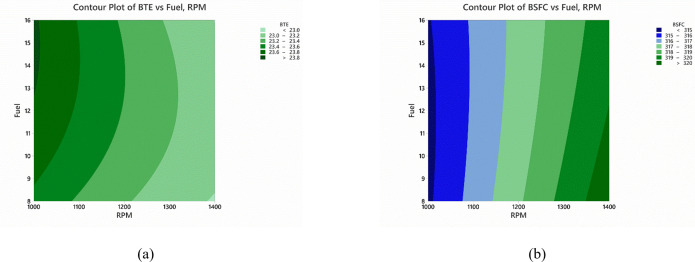
Contour Plots for the (a) BTE (b) BSFC using RSM for Heavy Duty Industry.

The regression model was generated using the full factorial experimental study is shown Table 13. The contour plots were made to have better understanding of the results. Fig 9 demonstrates the contour plot for BTE under the effect of biodiesel fuel percentage and engine speed simultaneously. The plot demonstrates that the decrease in the biodiesel content in fuel with low-speed operations, the maximum value of efficiency can be achieved. On the other hand, Fig 9 demonstrates that the least BSFC can be obtained at low speeds but with higher biodiesel content. This is due to low HHV of the biodiesel fuel in comparison to diesel, thus requiring more power for combustion of the fuel to generate same amount of energy without impacting the conversion efficiency of the engine. This alone proves that the biodiesel content should be added to a limited range in the engine.

The slightest of changes would result in higher frictional losses as well as more wear in the engine. Thus, in the longer run, smaller amounts of biodiesel mixture could be not only economical in use but also bearable for the engine without affecting its longevity [73]. The better operational zones for the performance of the engine can be identified based on the contour plots in the Fig 9. Therefore, a few cases demonstrated for potential improvement in the operation at higher speed are listed in the Table 15.

**Table 15:** Selected Case settings for the optimal Engine performance for Heavy Duty industry.

Case	Adverse Settings	BTE %	Selected Settings	BTE %	% Savings
Maximize BTE	Fuel~12.0 Speed~1000 Load~75 MgO ~ 0.04	23.58	Fuel~15.0 Speed~600 Load~75 MgO ~ 0.04	24.35	5%
Maximize BTE	Fuel~10 Speed~900 Load~75 MgO ~ 0.04	23.96	Fuel~13.0 Speed~650 Load~75 MgO ~ 0.04	24.50	5%

#### 3.4.2. Automotive industry.

**Fig 10:**
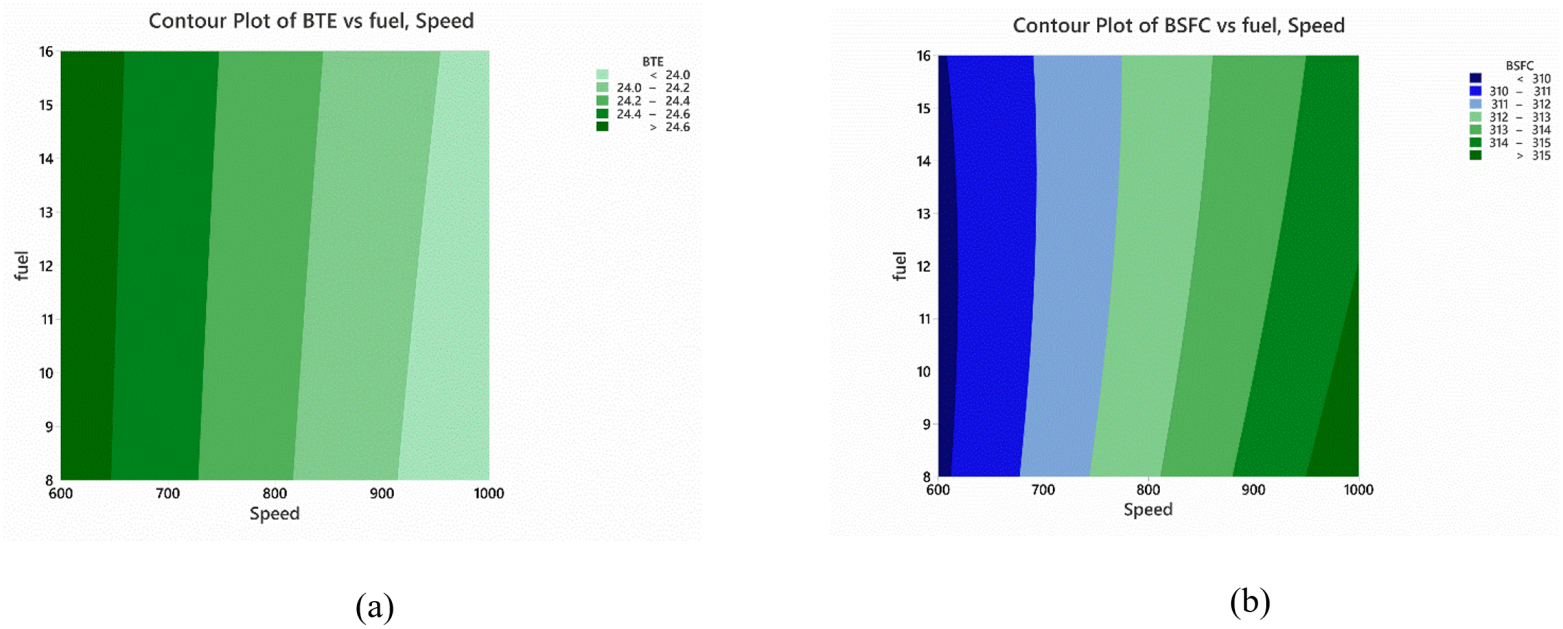
Contour Plots for the (a) BTE (b) BSFC using RSM for Automotive Industry.

Using the full factorial experimental design as shown in Table 14, a regression model was created. The effect of biodiesel fuel percentage and engine speed on the BTE is shown in the form of contour plot in Fig 10. The findings suggest that optimum power is delivered at low RPM with low biodiesel blend rates. On the other hand, it can be seen from the Fig 10 source that the desired biodiesel blend provided best specific fuel consumption. This may be due to the difference in HHV of biodiesel and diesel fuel.

Table 16 provides the optimal operational zones for the automotive industry. The zones were identified based on the contour plots shown in Fig 10. Overall savings of 6%−10% were identified and will impact on the financial decisions of the everyday consumers.

**Table 16:** Selected cases for automotive industry.

Case	Adverse Settings	BTE %	Selected Settings	BTE %	% Savings
Maximize BTE	Fuel~10.0 Speed~2000 Load~75 MgO ~ 0.04	21.7	Fuel~16.0 Speed~1400 Load~75 MgO ~ 0.04	23.1	6%
Maximize BTE	Fuel~15 Speed~2000 Load~75 MgO ~ 0.04	21.6	Fuel~14.0 Speed~1200 Load~75 MgO ~ 0.04	23.5	7%
Maximize BTE	Fuel~16.0 Speed~1800 Load~75 MgO ~ 0.04	22.1	Fuel~16 Speed~10070 Load~0 MgO ~ 0.04	24.0	7%

### 3.5. Post combustion nanoparticle management

The inclusion of MgO nanoparticles has significantly improved BTE and reduced BSFC but it is significant to keep an eye on the environmental and health implications of the nanoparticles used in engines. A small fraction of nanoparticles may remain unburned or partially combusted [74]. To mitigate the spread of nanoparticles in the environment post combustion nanoparticles management strategies are utilized. Following strategies are recommended to reduce the health and environmental risks:

- Electrostatic precipitators are widely used to capture fine and ultrafine particles by applying a high-voltage electric field that charges particles and collects them on oppositely charged plates. In the study by De Oliveira et. al. [75], an ESP integrated diesel engine running on carbon nanotube – enhanced fuels reduced nanoparticles emissions by up to 90%.
- Diesel particulate filters utilize the porous ceramic substrates trap soot and nanoparticles in wall-flow configurations. Lee et. al. [76] showed that the DPFs with the nano-biodiesel blends could retain more than 80% of emitted metal oxides.
- Catalytic converters coated with metal oxides or zeolite transform the residual nanoparticles into the less harmful compounds. Navarro-Espinoza et. al. [77] emphasized the need for catalytic oxidation of MgO and ZnO residues in nanoparticle – enhanced fuel systems to ensure regulatory compliance.

Recent developments involve coupling ESPs with plasma assisted oxidation systems for heavy duty engines. Therefore, while MgO nanoparticle addition boosts combustion and performance, its practical deployment must be paired with appropriate nanoparticle technologies to meet the Euro VI emissions. Improvement in the BTE and BSFC indicate that enhanced combustion completeness and improved fuel utilization. Several studies on metal – oxide nano – additives report such performance improvements often correspond to the lower CO and HC due to more complete oxidation, while NO_x_ trends depends upon peak cylinder temperatures and oxygen availability [78].

## 4. Conclusion

The study demonstrated the utilization of the Taguchi based RSM method for the optimization of the performance metrics of the diesel engine. Using L18 orthogonal array, the effects of nanoparticle concentration, engine load, and blend ratio were systematically studied with reduced number of experiments, ensuring statistical robustness and operational efficiency. The study successfully utilized the Taguchi experimental design and full factorial design approaches for the optimization of the diesel engine running on the biodiesel-diesel blends and MgO nanoparticles. The optimum operational parameters for the Diesel engine utilizing biodiesel and MgO as nanoparticles were identified. MgO was proved to be essential along with the Biodiesel to provide best efficiency for the engine. Lower RPM settings were identified as another key operational parameter for the optimum performance of engine. The conclusions drawn from the current research work are follow:

- Stepwise forward regression (SFR) proved effective in identifying key input-output relationships, outperforming the models like general linear model (GLM) and linear regression (LR). The addition of the biodiesel at 12–16% resulted in the significant improvement in BTE. Although blends exceeding the 16% biodiesel caused a reduction in BTE due to the biodiesel’s lower calorific value and other combustion challenges. MgO nanoparticles improved the combustion efficiency, leading to high BTE.
- Speed was considered as the critical decision factor for the automotive and heavy machine industry. Lower speed operation from 600RPM to 1000 RPM was important for the heavy machine industry while the speed in the range of 1000 RPM to 1400 RPM was significant for the optimal automotive engine performance.
- Response surface for the speed with BTE for the heavy machine industry shows the quadratic relation. At low RPMs, with the load of 75%, biodiesel percentage under 16% and 0.04 g MgO in fuel, the optimal BTE can be obtained. Typically, there was 5% savings for the selected settings for the engine operation as compared to the adverse settings.

RSM for the automotive industry indicated that the speed has quadratic relation with the engine performance. At RPMs ranging between 1200 and 1400 along with the high biodiesel ratio of 16%, Load of 75% and 0.04 g MgO, up to 7% increase in the BTE was observed. This work is limited to the performance optimization. For full environmental and operational evaluation, we will perform a detailed combustion and emission campaign at the optimized operating points.

Considering these observations, following future research directions are proposed:

- Investigation of the potential corrosive or abrasive effects of nano particles on the fuel lines, nozzles and filters,
- Full techno economic evaluation should be conducted to assess the viability of nanoparticle usage in commercial-scale biodiesel blending.
- Life cycle Assessment of the utilization of the nano particles-based biodiesel blends need to be performed for overall environmental effects.
- Combining this system with the diesel particulate filters and exhaust gas recirculation systems could offer more holistic approach to emission control.
- Planned diagnostics including cylinder pressure, exhaust gas measurements, particulate mass and number concentration, and long engine durability tests to examine deposits and wear in the engine fuel line and emission line.

**Table:** 

Nomenclature	
BTE	Brake Thermal Efficiency
BSFC	Brake Specific Fuel Consumption
CI	Compression Ignition
DI	Direct Injection
DOE	Design of Experiments
GLM	General Linear Model
HHV	Higher Heating Value
MgO	Magnesium Oxide
NPs	Nanoparticles
RSM	Response Surface Methodology
RPM	Revolutions Per Minute
SFR	Stepwise Forward Regression
**Symbols**	
*η*ₜ	Thermal Efficiency (%)
*η*ₘ	Mechanical Efficiency (%)
*η*ₛ	Volumetric Efficiency (%)
*BSFC*	Brake Specific Fuel Consumption (g/kWh)
*BTE*	Brake Thermal Efficiency (%)
*P*	Power Output (kW)
*CV*	Calorific Value (kJ/kg)
*ρ*	Density (kg/m³)
*ν*	Kinematic Viscosity (mm²/s)
*Fr*	Frictional Losses (N·m)
*ω*	Angular Speed (rad/s)
*τ*	Torque (N·m)
*λ*	Air-Fuel Equivalence Ratio
*T*	Temperature (°C)
*Pb*	Brake Power (kW)
${\dot{m}}_{f}$–	Mass Flow Rate of Fuel (kg/h)
${\dot{m}}_{a}$–	Mass Flow Rate of Air (kg/h)
*CN*	Cetane Number
*F/A*	Fuel-to-Air Ratio

## Supporting information

S1 File Supplementary data table. (PDF)
