# Supplementary material for: Pairing taguchi based design of experiment with response surface methodology for diesel engine performance optimization using biodiesel-magnesium oxide nanoparticles blends
Source: PLoS One. 2026 Mar 4;21(3):e0341542. doi: 10.1371/journal.pone.0341542 (PMC12959659; doi:10.1371/journal.pone.0341542)
Supplement: S1 File — (PDF) [file pone.0341542.s001.pdf]

The data provided in the table below acts as the Supporting information data for the paper.

Table 1Supplimentary data table

**Experiment Number**	**A**	**B**	**C**	**D**	**BSFC**	**BTE**
	**Speed**	**Load**	**Fuel**	**MgO**		
	**RPM**	**%**	**Biodiesel %**	**g**	**g/kW**	**%**
**1**	1200	25	0	0	319.00	18.50
**2**	1600	50	0	0.02	327.45	20.54
**3**	2000	75	0	0.04	332.61	21.48
**4**	1200	25	4	0.02	317.00	20.80
**5**	1600	50	4	0.04	316.00	20.94
**6**	2000	75	4	0	329.77	21.07
**7**	1200	50	8	0	320.94	21.24
**8**	1600	75	8	0.02	323.45	22.17
**9**	2000	25	8	0.04	313.00	19.20
**10**	1200	75	12	0.04	318.06	23.27
**11**	1600	25	12	0	313.00	19.40
**12**	2000	50	12	0.02	340.00	20.24
**13**	1200	50	16	0.04	324.00	22.04
**14**	1600	75	16	0	342.47	22.17
**15**	2000	25	16	0.02	304.00	18.70
**16**	1200	75	20	0.02	310.00	23.27
**17**	1600	25	20	0.04	315.00	20.00
**18**	2000	50	20	0	350.00	18.30
